# Uncertainty Quantification in Inverse Scattering Problems

**DOI:** 10.3390/e28040461

**Published:** 2026-04-17

**Authors:** Carolina Abugattas, Ana Carpio, Elena Cebrián

**Affiliations:** 1 Facultad de Ingeniería, Universidad Alberto Hurtado, Santiago de Chile 8340575, Chile; cabugattas@uahurtado.cl; 2 Departamento de Análisis Matemático y Matemática Aplicada, Universidad Complutense, 28040 Madrid, Spain; 3 Departamento de Matemáticas y Computación, Universidad de Burgos, 09001 Burgos, Spain; elenac@ubu.es

**Keywords:** Bayesian inversion, uncertainty quantification, Markov chain Monte Carlo, Karhunen–Loève expansions, geophysical imaging, medical imaging

## Abstract

Inverse scattering problems seek anomalies in a medium given data measured after the interaction with emitted waves. Due to noise, predictions about the nature of these inclusions should be complemented with uncertainty estimates. To this end, we propose a progressive framework for inverse scattering from low- to high-dimensional Bayesian formulations depending on the prior information and the problem complexity. We aim to reduce computational costs by exploiting educated prior information. When we look for a few well-separated inclusions in a known medium with information about their number, we resort to low-dimensional parameterizations in terms of a few random variables representing their shape and material constants. We test this approach detecting anomalies in tissues and deposits in stratified subsoils. In more complex situations where the anomalies may overlap, we propose high-dimensional parameterizations obtained from Karhunen–Loève (KL) or Fourier expansions of the density and velocity fields. We employ these methods to characterize oil and gas reservoirs in a salt dome configuration, where the screening effect of the dome cap prevents the obtention of adequate prior information. We characterize the posterior probability by means of affine invariant ensemble and functional ensemble MCMC samplers depending on dimensionality. This provides information on configurations with the highest a posteriori probability and the uncertainty around them, identifying factors that could reduce the uncertainty. In high-dimensional setups, techniques based on KL developments are more effective and stable. A recurring issue is the choice of the a priori covariance (which strongly affects the results) and the choice of its hyperparameters. Here, we use educated choices. Formulations that include them as additional parameters could be a next step at a higher cost.

## 1. Introduction

Subsurface structures are typically inspected by imaging techniques based on wave emission and recording at specific sites. Depending on the waves employed we have different imaging techniques. We focus here on quantifying uncertainty in imaging approaches that rely on elastic waves, with applications in medical and geophysical imaging.

Geophysical imaging methods include electrical resistivity tomography, ground-penetrating radar, induced polarization seismic tomography, reflection seismology and magnetotellurics. Seismology, for instance, estimates subsurface properties from elastic energy created by natural and artificial sources [1]. The technique requires a controlled source of elastic waves, such as a seismic vibrator, an explosion, or an air gun in marine data acquisitions. Seismic waves are often modeled as one-dimensional rays, a method referred to as ray theory, which can fit travel time data well through reflection and migration techniques [2,3]. However, the full waveform contains more information than just the travel times, which has led to the development of tomography methods [4]. In geophysical imaging, there are multiple sources of uncertainty, such as the huge size of the regions to explore, multiple reflections and distortions due to the heterogeneity and change in rock type, fluctuations in the terrain and other causes of noise. Therefore, we need to be able to quantify the uncertainty caused by such fluctuations in our predictions.

Medical imaging aims to visualize internal tissue structures by non-invasive techniques [5]. Popular methods include X-ray radiography, nuclear medicine, magnetic resonance imaging, ultrasound, echography, tomography, endoscopy, elastography, thermography, and holography. Elastography is an imaging technique based on the elastic properties of soft tissue [6,7]. Healthy tissue is often softer than cancerous tumors (breast and prostate tumors, for instance, see [7,8,9]). Similarly, healthy liver tissue is softer than damaged liver tissue. Existing technology [10] can distinguish healthy and unhealthy tissue in some situations. However, the imaging of tissues containing tiny tumors, multiple anomalies, or blurry regions with little contrast is strongly affected by noise and fluctuations. Uncertainty due to different factors needs to be quantified.

Characterizing the structure of reservoirs from full waveform seismic data or the structure of tissues from elastography data are typical examples of inverse problems. Knowing the signal emitted at the sources and the measurements recorded at sets of receivers, we aim to describe the structure and composition of a medium. Inverse problems arise in many other contexts (non-destructive testing of materials and structures, security, etc.) and are severely ill posed [11]. In spite of the vast number of techniques created so far, there is a need for ever-better methods. Specifically, developing uncertainty quantification tools to assess the effect of noise and fluctuations in the predictions is an active field of research nowadays. In this line, Bayesian inversion approaches are a promising tool to handle inverse scattering problems while quantifying uncertainty [12,13,14]. In the Bayesian framework, noise, unknowns and model parameters are represented as random variables or fields. Our previous knowledge of the solution acts as a regularizing factor through an a priori distribution. The solution to a Bayesian inverse problem is the posterior distribution, that is, the conditional distribution of the unknowns given (i) the prior density of the unknowns, which incorporates prior (expert) knowledge and (ii) the likelihood or conditional probability of the observing the data given the variables according to a certain model of wave propagation. Defining a likelihood requires the choice of a metric. Euclidean distances weighted with a covariance matrix are typical choices; see [15] for a review and for recent developments such as entropy-based metrics. The configurations with the highest probability (maximum a posteriori estimates) are candidate solutions to the original inverse problem, with uncertainty characterized by the posterior distribution. Often, the posterior distributions can be approximated by expressions of the form $e^{-J(\nu )}$, where ν represents the unknown variables or fields. Then, the maximum a posteriori (MAP) estimates are solutions of constrained optimization problems where the equations for wave propagation act as a constraint, which can be solved by Newton and Levenberg–Marquardt type techniques [16,17]. The functional J(ν) is usually given by the metric employed in the likelihood plus a regularizing term that uses the a priori information. Purely deterministic approaches typically aim to optimize alternative constrained functionals that employ a variety of Tikhonov and total variation regularization terms [17,18,19]. Deterministic methods provide a solution for each dataset considered, that can even be robust for very small noise under specific conditions when the material nature is known [17,18]. However, they do not assess how those configurations change with increasing noise levels and what is their range of variability. In the Bayesian context, the MAP estimates provide the most likely anomaly configuration at a moderate computational cost. Resorting to a Laplace approximation, one can quantify uncertainty to a certain extent. This approximation linearizes the posterior distribution about the MAP estimate [20] and replaces the posterior with a Gaussian distribution. Asymmetry and multimodality features are lost [21]. When the MAP point and the mean of the posterior distribution agree, generalized stochastic perturbation techniques [22] may provide a way to obtain information on skewness and other features. However, posterior distributions often encode more information that the MAP estimate and simple approximations. Sampling the posterior distribution by means of Markov chain Monte Carlo (MCMC) methods we can obtain a precise characterization, including the potential presence of several high probability configurations and asymmetry effects in the tails. MCMC sampling is an optimization technique itself: it provides all the minima of the cost J(ν), ranked according to the value of the cost (i.e., their probability), but it also gives information on the uncertainty around these values through sample processing.

A wide variety of strategies to generate Markov chains to sample from target probability distributions are available: Metropolis–Hastings (MH), Hamiltonian Monte Carlo (HMC), preconditioned Crank–Nicholson (pCN), ensemble affine invariant samplers (AIES), ensemble pCN based samplers and functional ensemble based samplers (FES); see [21] for a review and references therein. Compared to MH methods, HMC methods reduce correlations in the samples. The standard HMC algorithm is inherently sequential due to its trajectory-building steps and tends to get trapped around a single mode when sampling multimodal distributions. However, there are recent parallelization attempts [23]. Instead, AIES are designed to be parallelizable and are known to be efficient for poorly scaled and/or multimodal distributions in low to moderate dimensions [24]. As the dimension increases, these methods present vanishing acceptance rates. pCN methods were introduced to overcome this issue, displaying robust acceptance rates as the dimension tends to infinity [14]. Nevertheless, they are slow for poorly scaled or multimodal distributions. Combinations of pCN and AIES such as FES are robust as dimensions tend to infinity, parallelizable, gradient free, and can be designed to handle poorly scaled or multimodal distributions [25]. Since we face poorly scaled and multimodal distributions in increasing dimensions, AIES and FES seem to be well adapted to sample the posterior distributions we will consider. Parallelization is an additional asset since each likelihood evaluation involves solving an initial boundary value problem by hybrid finite difference–finite element techniques, which is computationally quite expensive.

To frame the problems of subsurface characterization in a Bayesian inversion context, we must choose a mathematical representation of the unknown features and determine the a priori information that we can include. This leads to different approaches to the problem, whose computational cost may be prohibitive. In this work, we focus on situations where existing information about the subsurface (provided by other techniques) allows us to adopt low- or moderate-dimensional representations of the unknown subsurface elements. Under these conditions, we can formulate Bayesian versions of the inverse problem with a manageable computational cost. Developing cost reduction strategies, such as reduced-order models or deep learning surrogates [26,27,28] is an active research field nowadays. However, the applicability these methods to time dependent wave problems with dynamically changing spatiotemporal wave patterns is still a subject of research [29].

**Figure 1:**
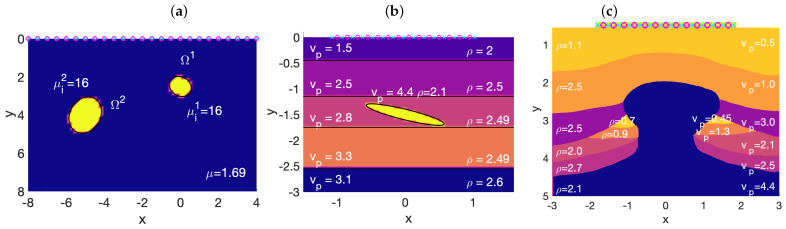
Scheme of typical imaging setups: (**a**) anomalies in tissues, (**b**) inclusions in stratified soil and (**c**) gas and oil reservoirs attached to salt domes. Units for shear moduli μ, densities ρ and velocities $v_{p}$ are kPa, $\frac{10^{3}\;\mathrm{kg}}{m^{3}}=\frac{g}{{\mathrm{cm}}^{3}}$ and $\frac{\mathrm{km}}{s}$. Parameter values correspond to healthy and scar liver tissue in (**a**) and to sandstone, limestone, shale and salt in (**b**,**c**), including gas and oil in (**c**). Circles and crosses represent emitters and receivers.

The paper is organized as follows. Section 2 formulates the general uncertainty quantification framework for the inverse problem under consideration. We detail the forward wave model in Section 3. Before proposing a Bayesian formulation, we need to decide how to represent mathematically the details of the subsurface structure that we ignore. In this context, they would be characterized by means of density, velocity and/or elastic moduli fields. In principle, we should determine these fields everywhere in the computational region, which is an infinite-dimensional problem. Finite-dimensional Bayesian formulations that are robust as the dimension grows are suggested in [12], for instance. They represent the unknown fields by their values at all the points of a fine mesh that covers the whole computational region. Here, we propose a progressive framework from low- to high-dimensional Bayesian formulations depending on the prior information, which relies mostly on bases expansions or specific parametrizations on the unknown geometry. We will consider two different situations of increasing complexity. In both, we assume we have some knowledge of the subsurface structure, obtained by other methods. We wish to determine the detailed composition of some regions, where we suspect the presence of inclusions of a different nature. In Section 4 we characterize the inclusions by a small number of parameters that define their shape and material constants. Then, we present uncertainty quantification tests in medical elastography and in geophysics based on AIES (that are known to be effective for poorly scaled problems and multimodal distributions in low dimensions), see Figure 1. Uncertainty tends to be larger in geophysical tests since they typically involve two material constants and stratified configurations, which favors the appearance of several high probability configurations. Section 5 characterizes inclusions in terms of perturbations of the a priori known density and velocity fields. Such perturbations are defined by means of series expansions. In principle this is an infinite-dimensional problem, that we approximate in finite dimensions by means of truncated expansions. We consider two types of parameterizations. The first one is defined by the coefficients of truncated Fourier series expansions, which becomes a finite set of random variables. The second one works with truncated Karhunen–Loève expansions [14,25,30] of the density and velocity random fields. We tests the methods on a salt dome geometry to evaluate the presence of possible oil/gas reservoirs. To sample the resulting posterior probability densities we resort to FES. As previously said, these samplers have a robust acceptance rate as the dimension tends to infinite, are parallelizable and have the potential of handling complex distributions. Finally, Section 6 discuses the results and summarizes our conclusions. A final appendix describes the MCMC ensemble sampling algorithm used, that adapts the original algorithm to work with several fields.

## 2. Bayesian Inverse Problem

Given a random field ξ, Bayes formula in infinite dimensions [14,31,32,33] states that the Radon–Nikodym derivative of the posterior probability measure $\pi_{\mathrm{pt}}$ with respect to the prior measure $\pi_{\mathrm{pr}}$ is given in terms of the conditional probability density function

(1) $$\begin{array}{c} \frac{d\pi_{\mathrm{pt}}}{d\pi_{\mathrm{pr}}}=p\left(d|\xi \right)\frac{d\pi_{\mathrm{pr}}}{I},\;I=\int \pi \left(d|\xi \right)d\pi_{\mathrm{pr}}, \end{array}$$

where $\pi_{\mathrm{pr}}$ is the prior distribution, p(d|ξ)=L(ξ) is the likelihood and d are the available data. In principle, our inverse problem would consist in characterizing this probability when ξ represents the underground density and velocity fields and d the data recorded in an imaging setup.

Dimensionality can be reduced in several ways so that the unknown density and velocity fields are approximated by a finite number of parameters. In this framework, such parameters ν are considered to be a finite set of random variables. According to Bayes’s Theorem [33]

(2) $$\begin{array}{c} p_{\mathrm{pt}}\left(\nu \right):=p\left(\nu |d\right)=\frac{p\left(d|\nu \right)p_{\mathrm{pr}}\left(\nu \right)}{p(d)}, \end{array}$$

where $p_{\mathrm{pr}}\left(\nu \right)$ is the prior probability of the parameters (that encodes available information or known restrictions), p(d|ν)=L(ν) is the conditional probability or likelihood (that reflects the fidelity to the measured data of the data that would be observed for ν as predicted by a model of wave propagation), and p(d) acts as a normalization factor. Different metrics can be selected to compare the measured data and the data predicted by a model in the likelihood definition (see [15] for a review and for new developments such as entropy-based metrics). Here, we mostly resort to Mahalanobis-type metrics, which are essentially Euclidean distances weighted with a covariance matrix. We will consider next two different kinds of finite-dimensional reductions depending on the a priori knowledge on the subsurface. First, we need to discuss the model of wave propagation selected to define the likelihood.

## 3. Physical Setups and Observation Operator

Typical setups locate a number of sources $x_{k},\;$k=1,…,K, at the upper surface that launch intense signals into the imaged medium. After interaction with subsurface structures, the waves are recorded at receivers $r_{j}$, j=1,…,J, for some time. The recorded signals constitute the data d and are functions of the wavefield values at the receiver sites. We describe wave dynamics by a simplified model of wave propagation through the subsurface

(3) $$\begin{array}{c} \begin{array}{c} \rho \left(x\right)u_{tt}-\mathrm{div}\left(\mu \left(x\right)\nabla u\right)=f\left(t\right)g\left(x\right),\;x_{2}<0,\;x_{1}\in R,\\ \frac{\partial u}{\partial n}\left(x_{1},0,t\right)=0,\;x_{1}\in R,\\ u\left(x,0\right)=0,u_{t}\left(x,0\right)=0,\;x_{2}<0,\;x_{1}\in R, \end{array} \end{array}$$

where $x=\left(x_{1},x_{2}\right)\in R^{2}$, t>0, and u(x,t) represents the scalar displacements in the (negative) vertical $x_{2}$ direction. The term f(t)g(x) describes wave emission at sources. The function *f* is a Ricker wavelet of frequency $f_{M}$ related to the spatial resolution expected and the distance between emitters/receivers $f\left(t\right)=f_{0}\left(1-2\pi^{2}f_{M}^{2}t^{2}\right)e^{-\pi^{2}f_{M}^{2}t^{2}}.$ The function *g* represents the perturbation created by the emitters, for instance, $g\left(x\right)=\frac{1}{{\left(\pi \kappa \right)}^{n/2}}\sum_{k=1}^{K}e^{\frac{-|x-x_{k}{|}^{2}}{\kappa }}$, where κ>0 is a regularizing parameter and n=2 the spatial dimension. It has zero normal derivative at the interface. In medical elastography, (3) describes the propagation of shear waves in the tissue and the coefficient μ represents its shear modulus. In geophysical imaging, (3) governs the longitudinal waves that reach deep underground and μ relates to densities and speeds by $\mu =\rho v_{p}^{2}$.

We truncate the half space to a finite rectangular domain *R* and impose non-reflecting boundary conditions at the artificial vertical and horizontal walls to approximate the solution of ((3), see [13,16]). In what follows, we will work with a non-dimensionalized version of the truncated model obtained by the change of variables: $x=x^{\prime }L$, $t=t^{\prime }T$, $u=u^{\prime }L$, $R=R^{\prime }L$, $\Sigma =\Sigma^{\prime }L$, $\rho =\rho^{\prime }\rho_{0}$ and $\mu =\mu^{\prime }\rho_{0}L^{2}/T^{2}$. Here, *T*, *L* and $\rho_{0}$ are characteristic time, length and density scales. Dropping the symbol ’ for ease of notation, we find

(4) $$\begin{array}{c} \begin{array}{cc} \rho u_{tt}-\mathrm{div}\left(\mu \nabla u\right)=\tilde{f}\left(t\right)\tilde{g}\left(x\right), & x\in R,\;t>0,\\ \frac{\partial u}{\partial n}=0, & x\in \Sigma ,\\ \frac{\partial u}{\partial n}=\frac{-1}{c}u_{t} & x\in \partial R\setminus \bar{\Sigma },\\ u\left(x,0\right)=0,u_{t}\left(x,0\right)=0, & x\in R. \end{array} \end{array}$$

Here, $\tilde{f}\left(t\right)=\frac{T^{3}}{\rho_{0}L}f\left(Tt\right)$ and $\tilde{g}\left(x\right)=g\left(Lx\right)$. Σ represents the upper boundary. The boundary condition on the artificial boundaries $\partial R\setminus \bar{\Sigma }$, with $c=\frac{\sqrt{\mu }}{\sqrt{\rho }}$, is non-reflecting as long as the velocity and density fields remain piecewise constant nearby and discontinuity curves are orthogonal to the boundary (see [16]). We will make this assumption on the imaged region. Well-posedness and stability results for this model have established in [13,16], where low-cost finite element (FEM) schemes are also discussed.

Given a model of wave propagation such as (4), we define a basic observation operator by recording wave profiles at the receivers $r_{j}$, j=1,…,J, for a sequence of times $t_{m}$, m=1,…,M. For each realization of the coefficient fields, the observation operator is

(5) $$\begin{array}{c} F:\left(\rho ,\mu \right)\to {\left(u_{\rho ,\mu }\left(r_{j},t_{m}\right)\right)}_{j=1,\ldots ,J,m=1,\ldots ,M} \end{array}$$

where $u_{\rho ,\mu }$ is the solution of (4). Depending on the nature of the receivers and the expected regularity of the wave field, other choices may be advisable, such as averages in small areas at each receiver location. In practice, we construct numerical solutions of (4) using the finite element method. Therefore, the observation operator (5) will use the numerical solution. For a study on the effect of numerical choices on the observation operator and the numerical solution of inverse Bayesian problems, we refer the reader to [16].

We assume that the measured data are related to the observation operator by

$$\begin{array}{c} d=F(\rho_{\mathrm{true}},\mu_{\mathrm{true}})+\eta , \end{array}$$

where $\rho_{\mathrm{true}}$, $\mu_{\mathrm{true}}$ represent the density and the velocity fields for the explored region and η is a noise term. To simplify, we assume Gaussian noise with zero mean and correlation $\Gamma_{n}$, that is, $\eta \in N(0,\Gamma_{n})$. In practice, this may not be the case and other probability distributions can be used [34,35].

## 4. Finite Random Variable Approach

In this section, we assume that we have information on the overall subsurface structure (obtained by other techniques) except for details on the presence of localized inclusions of different materials.

Each inclusion is parametrized in terms of a few parameters $\nu^{\left(k\right)}$. Concatenating them, the vector $\nu =(\nu^{\left(1\right)},\ldots ,\nu^{\left(L\right)})$ represents *L* possible inclusions. We define $p_{\mathrm{pr}}\left(\nu \right)$ in terms of a truncated multivariate Gaussian

(6) $$\begin{array}{c} p_{\mathrm{pr}}\left(\nu \right)=\left\{\begin{array}{cc} \frac{p_{0}}{{\left(2\pi \right)}^{N/2}}\frac{1}{\sqrt{\det(\Gamma_{\mathrm{pr}})}}\exp\left(-\frac{1}{2}{\left(\nu -\nu_{0}\right)}^{⊤}\Gamma_{\mathrm{pr}}^{-1}\left(\nu -\nu_{0}\right)\right), & \nu \in M,\\ 0, & \nu \notin M, \end{array}\right. \end{array}$$

where *N* is the dimension of $\nu_{0}$ and M incorporates a set of constraints on the coefficients. The symbol ⊤ denotes transposition. The number $p_{0}$ is a normalization constant, whose value is not needed in practice. The vector $\nu_{0}$ represents a priori information on the possible values of the sought parameters, while $\Gamma_{\mathrm{pr}}$ represents the covariance matrix. We define the likelihood as

(7) $$\begin{array}{c} p\left(d|\nu \right)=\frac{1}{{\left(2\pi \right)}^{D/2}\sqrt{\det(\Gamma_{n})}}\exp\left(-\frac{1}{2}{\left(F(\nu )-d\right)}^{⊤}\Gamma_{n}^{-1}\left(F\left(\nu \right)-d\right)\right), \end{array}$$

where $\Gamma_{n}$ is the covariance matrix for the noise affecting the data d. We chose here a D×D diagonal matrix, with constant diagonal given by $\sigma_{n}^{2}$. $F\left(\nu \right)$ represents the observation operator (5) when $\rho =\rho_{\nu }$ and $\mu =\mu_{\nu }$, that is, ρ and μ incorporate the presence of the inclusions defined by ν. Neglecting scaling factors, the posterior distribution (2) can be approximated by

(8) $$\begin{array}{c} p_{\mathrm{pt}}\left(\nu \right)\approx \exp\left(-\frac{1}{2}{\left(\nu -\nu_{0}\right)}^{⊤}\Gamma_{\mathrm{pr}}^{-1}\left(\nu -\nu_{0}\right)-\frac{1}{2}{\left(F(\nu )-d\right)}^{⊤}\Gamma_{n}^{-1}\left(F\left(\nu \right)-d\right)\right) \end{array}$$

for ν∈M and zero otherwise. The highest probability configurations minimize the regularized cost

(9) $$\begin{array}{c} J_{R}\left(\nu \right)=\frac{1}{2}{\left(\nu -\nu_{0}\right)}^{⊤}\Gamma_{\mathrm{pr}}^{-1}\left(\nu -\nu_{0}\right)+\frac{1}{2}{\left(F(\nu )-d\right)}^{⊤}\Gamma_{n}^{-1}\left(F\left(\nu \right)-d\right) \end{array}$$

for ν∈M.

### 4.1. Homogeneous Background

In homogeneous media, we can obtain educated a priori information on the number of non-overlapping inclusions *L*, their size and their location, by means of topological energies [13,36]. Iterative methods combining successive calculations of topological fields with deterministic optimization [18] can approximate with precision the geometry of anomalies in cases where the nature of the inclusions is known and noise levels are very small. However, it is quite difficult to predict the nature of the anomalies [36] due to the possible presence of additional local minima. Topological energy methods are robust to noise [37] in the sense that the number of predicted anomalies does not really change as we increase the noise levels. Capturing the right number of anomalies depends on factors such as the overall geometrical arrangement of the anomalies, the employed frequency, the region covered by the receivers and the distance between them, and the use of sweeping strategies (exploiting the finite speed of the waves to calculate topological energies with data from different fractions of receivers to cover different sectors) [13,38]. However, topological methods alone provide no insight on the nature of the inclusions. We can devise a procedure that uses part of the data to produce educated a priori information on the number and geometry of inclusions by topological methods, reserving the remaining amount of data for the Bayesian inversion problem in large noise contexts.

Let us assume that we have characterized the number of inclusions *L* by topological energy methods and extracted prior information on their geometry encoded in a set of parameters $\nu_{0}^{\left(\ell \right)}$, ℓ=1,…,L. Since we have no information on their nature, we set the a priori values for the material parameters equal to those of the surrounding medium. In the tests presented here, the number of objects predicted by topological energy methods remains the same if we vary the noise intensity: 5%, 15%, 20% …. Up to 70% noise the centers and sizes predicted by topological energy methods to construct the priors remain pretty similar too.

For simplicity, we choose starshaped parameterizations, whose radius is approximated by trigonometric polynomials. The contour of each inclusion $\Omega^{\ell }$ is given by

(10) $$\begin{array}{c} q^{\ell }\left(\theta \right)=\left(\nu^{\ell }\left(1\right),\nu^{\ell }\left(2\right)\right)+r^{\ell }\left(\theta \right)\left(\cos\left(2\pi \theta \right),\sin\left(2\pi \theta \right)\right),\;\theta \in \left[0,1\right], \end{array}$$

(11) $$\begin{array}{c} r^{\ell }\left(\theta \right)=\nu^{\ell }\left(3\right)+2\sum_{q=1}^{Q}\nu^{\ell }\left(2q+3\right)\cos\left(2\pi q\theta \right)+2\sum_{q=1}^{Q}\nu^{\ell }\left(2q+2\right)\sin\left(2\pi q\theta \right), \end{array}$$

and their material properties by $\nu^{\ell }\left(2Q+4\right)$, $\nu^{\ell }\left(2Q+5\right)$, for ℓ=1,…,L. The number of parameters N=L(2Q+5). While $\left(\nu^{\ell }\left(1\right),\nu^{\ell }\left(2\right)\right)$ represents the center, $\nu^{\ell }\left(3\right)$ is the first term of the radius expansion. Other parameterizations are possible (see [39], for instance). In this setup, the waves emitted by the sources are governed by (4) with

(12) $$\begin{array}{c} \rho \left(x\right)=\rho_{\nu }\left(x\right)=\left\{\begin{array}{cc} \rho , & x\in R\setminus {\bar{\Omega }}_{\nu },\\ \rho^{\ell }, & x\in \Omega^{\ell },\;\ell =1,\ldots ,L, \end{array}\right. \end{array}$$

(13) $$\begin{array}{c} \mu \left(x\right)=\mu_{\nu }\left(x\right)=\left\{\begin{array}{cc} \mu , & x\in R\setminus {\bar{\Omega }}_{\nu },\\ \mu^{\ell }, & x\in \Omega^{\ell },\ell =1,\ldots ,L, \end{array}\right. \end{array}$$

and $\Omega_{\nu }=\cup_{\ell =1}^{L}\Omega^{\ell }$. We choose a diagonal covariance matrix $\Gamma_{\mathrm{pr}}$ formed by *L* blocks. The components of each block corresponding to the radius must decay so that we favor positive radii and avoid cusps. We typically enforce power-like decay. This is reminiscent of deterministic inversion studies with star-shaped objects in other contexts, which use weighted metrics for the coefficients of the trigonometric polynomial defining the radius [17].

With these choices, we approximate the posterior distribution by (8). Since the number of parameters forming ν is usually small, and they are unbalanced, we sample (8) by means of an affine invariant ensemble sampler (AIES) (see [24]). This kind of Markov chain Monte Carlo samplers requires mixing W>2N chains to properly sample the distribution; see Appendix A for details.

**Figure 2:**
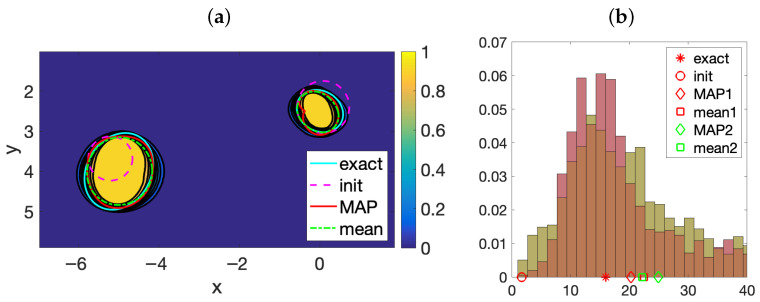
(**a**) Probability of belonging to the anomalies and (**b**) distribution of the shear modulus of the two anomalies (superimposed), constructed by AIES sampling of the posterior probability with liver tissue data. Anomalies are numbered from left (pink histogram, 1), to right (green histogram, 2).

Figure 2 illustrates the behavior of the posterior distribution (8) for a homogeneous tissue containing two anomalies, after 1000 steps of the MCMC algorithm implemented with parameters a=2 and W=480. In tissues, we can assume that $\rho =\rho_{0}$ everywhere. Thus, the parametrization of each object has only 2Q+4 components and N=L(2Q+4). The simulation employs data from liver tissue. Anomalies in the liver can represent scar tissue but also malignant tumors. Figure 2a represents the probability that a point belongs to an anomaly. The location, size and shape of the anomalies are well captured. The MAP and mean curves represent the highest probability contour and the contour corresponding to the average of the samples generated, excluding an initial subset discarded as a burn-in period. Figure 2b depicts the values for the shear moduli of the anomalies corresponding to each sample. Most of them lie around the true values (corresponding to scar tissue). However, the distribution has large tails that reach the healthy and malignant regions. These results correspond to data with 10% noise, and to prior information that is quite precise for locations and shapes, but it is blind to the nature of the anomalies. Whereas in healthy tissue μ∼1.69 kPa, in scar tissue $\mu_{s}∼16$ kPa, and in malignant tissues is much larger. We consider true inclusions formed by scar tissue, with $\mu_{i}∼\mu_{s}$, but keep μ as the a priori value for them in the posterior probability.

For this test, data have been generated synthetically, solving the forward wave problem (4) with the parameters listed in Table 1 and κ=2 in the geometry represented in Figure 1a. We locate transducers at fixed grids of step 0.5 on the upper border ranging from −12 to 12. Notice that the same devices act first as emitters and then as receivers; therefore, we can only record data once the emission is finished. Evaluating the numerical solution on a fixed time grid of step 0.025 in the time interval [2,11], we obtain the raw data $d_{j,\mathrm{true}}^{m}$, j=1,…,J, m=1,…,M. To generate the synthetic data, we add noise

(14) $$\begin{array}{c} d_{j}^{m}=d_{j,\mathrm{true}}^{m}+\varepsilon_{j},\;j=1,\ldots ,J,m=1,\ldots ,M, \end{array}$$

where ε is distributed according to $N(0,\Gamma_{n})$, with $\sigma_{n}=\alpha \;\max\left|d_{j,\mathrm{true}}^{m}\right|/100$. We set α=10, that is, 10 percent noise. The meshes we use to generate the synthetic data and evaluate the observation operator are different, with the first being finer. We split the data in two halves, corresponding to two interspaced grid with time step 0.05. We use one half to implement topological energy methods [13] to produce educated a priori information on the number of anomalies, their location, and size (dashed magenta circles in Figure 2a). However, we have no a priori information on the nature on the anomalies. We set $\nu_{0}^{\ell }\left(2Q+4\right)=\mu$, ℓ=1,2, equal to the shear modulus of the healthy tissue. As for the diagonal prior covariance, each block starts with ${\left(\sigma_{1}^{\ell }\right)}^{2}={\left(\sigma_{2}^{\ell }\right)}^{2}=0.1$ and ends with ${\left(\sigma_{2Q+4}^{\ell }\right)}^{2}=20^{2}.$ Then, ${\left(\sigma_{3}^{\ell }\right)}^{2}=0.1$ and ${\left(\sigma_{2q+2}^{\ell }\right)}^{2}={\left(\sigma_{2q+3}^{\ell }\right)}^{2}=0.1/{\left(1+q^{2}\right)}^{s}$, 1≤q≤Q, *s* large, as in [40], so that the prior favors regular shapes with r(t)>0. We fix s=3 and Q=5. Notice that $\sigma_{2Q+4}=20$ is a rather large value. The large deviation allowed in the prior would allow us to infer malignity if necessary.

If we include in the prior distributions more anomalies that needed, some of their shear parameters after sampling may essentially agree with the background medium, suggesting healthy tissue. If we include less anomalies than needed, we may encounter distributions with additional modes, associated to samples that capture the missing anomalies. Multimodality is often a consequence of insufficient prior information. As said before, being able to capture the right number of anomalies *L* by topological methods depends on the design of the imaging set-up (anomaly/detector arrangement, frequency choice) and the use of sweeping strategies that fraction the data to compute topological energies covering different regions. Inclusions can be missed if they are screened by other inclusions placed between them and the detectors or their size is below the employed imaging set-up resolution. Methods like the Bayesian Information Criterion (BIC) and Akaike Information Criterion (AIC) [41] are often employed to determine the most likely number of parameters to include in a Bayesian formulation. However, they do not provide a priori values for the centers of the anomalies and their geometrical parameters, so it is not clear how to implement them in this context. Ref. [40] explores a hierarchical Bayesian formulation with model selection through the model evidence to determine the number of anomalies *L* one should include in the formulation. However, it can only be used to choose between smaller numbers than detected by topological energies.

Topological energy methods would detect overlapping inclusions as one. They are typically employed when small sets of separated anomalies are expected. Since we are assuming here that each inclusion is characterized by constant material parameters we cannot study such anomalies within this framework. Section 5 proposes a different approach applicable to that context.

### 4.2. Stratified Background

In geophysics, we often work in stratified media. Topological energy tools are hardly useful in this context to obtain educated priors. Instead, we rely on prior information produced by other imaging techniques such as migration analyses [42]. To have a clear idea of the dimensions and orientation of the inclusions, we consider elliptic parameterizations $\nu^{\ell }$, where $\left(\nu^{\ell }\left(1\right),\nu^{\ell }\left(2\right)\right)$ represents the center; $\nu^{\ell }\left(3\right)$ and $\nu^{\ell }\left(4\right)$ the x and y semi-axes (respectively); and $\nu^{\ell }\left(5\right)$ the angle of the *x* semiaxis with the *x* axis. The last two parameters, $\nu^{\ell }\left(6\right)$ and $\nu^{\ell }\left(7\right)$, represent the density and the velocity, respectively. The total number of parameters is N=7L.

**Figure 3:**
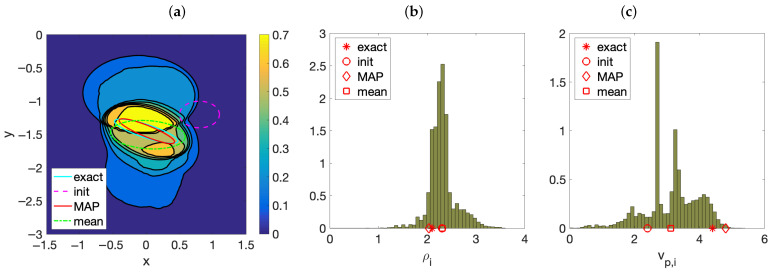
(**a**) Probability of belonging to the anomalies and (**b**,**c**) distribution of the density and the velocity in the inclusion, constructed by AIES sampling of the posterior probability with stratified soil data.

Figure 3 illustrates the behavior of the posterior distribution (8) for stratified media containing one inclusion, after 500 steps of the AIES algorithm, implemented with parameters a=2 and W=480. Figure 3a represents the probability that a point belongs to an anomaly. While the MAP estimate of its boundary is close to the true contour, the mean estimate differs noticeably. There is a broad region about the true inclusion formed by points with a relevant probability of belonging to the inclusion. The posterior probability happens to be multimodal, with families of samples accumulating around different shapes. In addition to the MAP estimate, that is, the curve of highest probability, there are secondary contours with high probabilities. The histograms in panels (b) and (c) visualize the distributions of densities and velocities of the samples. The densities concentrate around the true values and the MAP estimate for the density is very close to the true density. However, the distribution of the velocities is clearly multimodal. The MAP estimate is close to the true velocity, but the mean is just an average of this value and secondary high probability velocities (corresponding to the secondary high probability curves). Notice that this test employs a single frequency. When we combine several frequencies, the secondary modes dilute and the mode representing the true inclusion becomes dominant: uncertainty should diminish.

For this test, data have been generated synthetically, solving the forward wave problem (4) with the parameters listed in Table 2 and κ=0.04, in the geometry represented in Figure 1b. We locate the emitters and the receivers at interspaced grids of step 0.04: K=51 sources distributed in [−1,1] and J=52 detectors ranging from −1.02 to 1.02. Evaluating the numerical solution on a fixed time grid of step 0.1 in the interval [0,2.5], we obtain the raw data $d_{j,\mathrm{true}}^{m}$, j=1,…,J, m=1,…,M. To generate the synthetic data, we add noise according to (14) where ε is distributed following $N(0,\Gamma_{n})$, with $\sigma_{n}=\alpha /100\;{\left(\sum_{j=1}^{J}\sum_{m=1}^{M}\frac{|d_{j,\mathrm{true}}^{m}{|}^{2}}{JM}\right)}^{\frac{1}{2}}$. We set α=10, that is, 10 percent noise. The meshes we use to generate synthetic data and to evaluate the observation operator are different, the first one being finer. As before, we choose a diagonal covariance matrix $\Gamma_{\mathrm{pr}}$ formed by *L* blocks, *L* being the number of expected inclusions. The last two components in each block, that is, the standard deviations $\sigma_{\rho }$ and $\sigma_{v_{p}}$, are set equal to $\sigma_{\rho }=\left(\rho_{\max}-\rho_{\min}\right)/2$ and $\sigma_{v_{p}}=\left(v_{p,\max}-v_{p,\min}\right)/2$, see [16]. For the tests presented here we have set $\Gamma_{\mathrm{pr}}=$ diag (1, 1, 0.5, 0.5, 0.1, $\sigma_{\rho }^{2}$, $\sigma_{v_{p}}^{2}$). We assume we have a priori information on the location and size of the anomaly, provided by migration methods, but not on its nature. We select as prior means for ρ and $v_{p}$ the average of the minimum and maximum values for the layers: $\nu_{0}^{\ell }\left(6\right)=\left(\rho_{\max}+\rho_{\min}\right)/2$ and $\nu_{0}^{\ell }\left(7\right)=\left(v_{p,\max}+v_{p,\min}\right)/2$. This is a blind choice that does not introduce any specific information on the possible nature of the inclusions.

## 5. Random Field Approach

The previous section focuses on the detection of inclusions in a known medium, given information about their number but not their nature. We were able to estimate their material constants and improve the description of their geometry, with quantified uncertainty. When the number *L* of inclusions is unknown, it can be considered as another random variable of a discrete nature. The treatment of this type of formulations is complex and has been addressed, for example, in [40]. In this section, we choose to consider the material coefficients as random fields, whose peaks would locate inclusions of diverse nature, without neither imposing their presence, nor introducing information about their shape or location. We assume here that we have reliable information on the background stratified structure, excluding some localized regions which can contain deposits of different materials.

Figure 1c represents a geological structure of particular interest: the salt dome [43]. These domes form when underground salt (less dense than other rocks) moves slowly upwards, deforming and breaking up rocks in the way. These structures often mark the presence of oil-gas traps, when the combination of reservoir and source rocks around them is favorable (a source layer covered by a permeable reservoir rock layer lies under a low permeability cap layer). Salt layers attenuate and distort seismic signals, resulting in poor vertical resolution of standard techniques, as well as poor velocity maps [44]. The deposits are usually found in carbonate rocks, heterogeneous and with unknown fissure patterns, distribution and connection of pores. The contrast between rock types introduces multiple reflections. Recent work discusses the potential of FWI techniques [45,46,47] to characterize such reservoirs and underlines the relevance of noise effects in deposit characterization [48] (see also [49] for machine learning methods). We develop here alternative Bayesian approaches, assuming we have information (obtained by other techniques) on the overall subsurface stratified structure, except for the region located under the dome cap, which hides the oil and gas deposits.

We express the velocity and density fields as the sum of known background profiles $\rho_{0}$ and $v_{p,0}$ plus correctors $\rho_{c}$ and $v_{p,c}$:

(15) $$\begin{array}{c} \begin{array}{cc} \rho (x,y) & =\rho_{0}\left(x,y\right)+\rho_{c}\left(x,y\right),\\ v_{p}\left(x,y\right) & =v_{p,0}\left(x,y\right)+v_{p,c}\left(x,y\right), \end{array} \end{array}$$

which we expect to be relevant in the dome area. We propose next two possible formulations.

### 5.1. Fourier Series

Any square integrable two-dimensional function *h* admits a Fourier expansion converging to *h* in $L^{2}$, and converging also pointwise where continuous [50,51]. Assuming $\rho_{c}\in L^{2}\left(R\right)$, we approximate $\rho_{c}\left(x,y\right)$ in $R=\left[0,L_{x}\right]\times \left[0,L_{y}\right]$ by a truncated Fourier expansion

(16) $$\begin{array}{c} \rho_{c}\left(x,y\right)\approx \frac{A_{00}}{4}+\sum_{q=1}^{N_{x}}\left[\frac{A_{q0}}{2}\cos\left(\frac{2q\pi x}{L_{x}}\right)+\frac{B_{q0}}{2}\sin\left(\frac{2q\pi x}{L_{x}}\right)\right]\\ +\sum_{r=1}^{N_{y}}\left[\frac{A_{0r}}{2}\cos\left(\frac{2r\pi y}{L_{y}}\right)+\frac{B_{0r}}{2}\sin\left(\frac{2r\pi y}{L_{y}}\right)\right]+\\ \sum_{q=1}^{N_{x}}\sum_{r=1}^{N_{y}}\left[A_{qr}\cos\left(\frac{2q\pi x}{L_{x}}\right)\cos\left(\frac{2r\pi y}{L_{y}}\right)+B_{qr}\sin\left(\frac{2q\pi x}{L_{x}}\right)\sin\left(\frac{2r\pi y}{L_{y}}\right)\right]+\\ \sum_{q=1}^{N_{x}}\sum_{r=1}^{N_{y}}\left[C_{qr}\cos\left(\frac{2q\pi x}{L_{x}}\right)\sin\left(\frac{2r\pi y}{L_{y}}\right)+D_{qr}\sin\left(\frac{2q\pi x}{L_{x}}\right)\cos\left(\frac{2r\pi y}{L_{y}}\right)\right]=\rho_{\nu_{\rho }}\left(x,y\right). \end{array}$$

We relabel the coefficients as

(17) $$\begin{array}{c} \begin{array}{c} \nu_{\rho }=(A_{00},A_{1,0},\ldots ,A_{N_{x},0},B_{1,0},\ldots ,B_{N_{x},0},A_{0,1},\ldots ,A_{0,N_{y}},B_{0,1},\ldots ,B_{0,N_{y}},\\ \;A_{1,1},\ldots ,A_{1,N_{y}},\ldots ,A_{N_{x},1},\ldots ,A_{N_{x},N_{y}},B_{1,1},\ldots ,B_{1,N_{y}},\ldots ,B_{N_{x},1},\ldots ,B_{N_{x},N_{y}},\\ \;C_{1,1},\ldots ,C_{1,N_{y}},\ldots ,C_{N_{x},1},\ldots ,C_{N_{x},N_{y}},D_{1,1},\ldots ,D_{N_{x},N_{y}},\ldots ,D_{N_{x},1},\ldots ,D_{N_{x},N_{y}}), \end{array} \end{array}$$

and perform a similar procedure for $v_{p,c}$. In this way, we devise a finite-dimensional approximation to the correctors in (15), which becomes

(18) $$\begin{array}{c} \begin{array}{cc} \rho_{\nu } & =\rho_{0}+\rho_{\nu_{\rho }},\\ v_{p,\nu } & =v_{p,0}+v_{p,\nu_{v}}, \end{array} \end{array}$$

in terms of the parameters $\nu =(\nu_{\rho },\nu_{v})$, where $\nu_{\rho }$, $\nu_{v}$ represent the coefficients of the expansions (16) for $\rho_{\nu_{\rho }}$ and $v_{p,\nu_{v}}$, respectively. We set the total number of coefficients $N=2(1+2N_{x}+2N_{y}+4N_{x}N_{y})$ and use F(ν) to denote the observation operator (5) corresponding to the fields (18) with $\mu =\rho v_{p}^{2}$.

These coefficients constitute a set of random variables for which we define the posterior probability (2) according to Bayes’s Theorem. Assuming we have no information on the presence of traps around the dome, we select a truncated multivariate Gaussian prior distribution $p_{\mathrm{pr}}$ with zero mean $N(0,\Gamma_{\mathrm{pr}})$ and covariance $\Gamma_{\mathrm{pr}}$ when $\rho_{\nu }>0$ and $v_{p,\nu }>0$, and set $p_{\mathrm{pr}}\left(\nu \right)=0$ otherwise. Working with Fourier expansions, the only a priori information we have is that the series expansions should converge. Therefore, the coefficients must tend to zero in a way that their $\ell^{2}$ norm converges. Observing the coefficients of the Fourier series expansions of the type of fields involved in the study, we have not been able to obtain additional information on possible correlations. Therefore, we just chose a diagonal covariance matrix in which the entries corresponding to the modes (q,r) are given by $\frac{\sigma_{0}^{2}}{{\left(1+r^{2}+q^{2}\right)}^{s}}$, s>1. We estimate the value of $N_{x}$ and $N_{y}$ by approximating piecewise constant functions in the computational region on the computational mesh. With these choices, the likelihood is given by (7) and the posterior distribution to be analyzed is given by (8), with $\nu_{\rho }$ and $\nu_{v}$ defined by (16).

**Figure 4:**
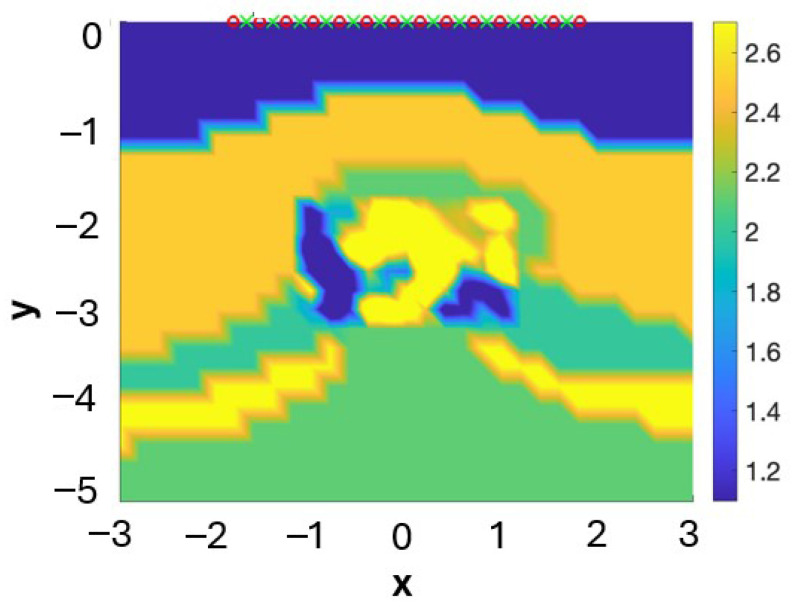
Density field ρ for the geometry in Figure 1c after 1500 steps of the AIES algorithm using a prior information that excludes the presence of oil and gas reservoirs. The image suggests the presence of reservoirs at the sides, under the dome cover.

We have employed the affine invariant ensemble sampler AIES [24] to characterize (8) for the salt dome problem. These methods do not construct a single chain but *W* chains that are mixed at each step. The method requires W>2N to work properly [24]. However, it is known that the chain mixing process becomes inefficient as dimensionality grows. It has been established that AIES methods are expected to be efficient to sample distributions involving small amounts of parameters, typically lower than 100 [24]. In our tests, we set $N_{x}=N_{y}=10$. The resulting number of total parameters *N* is rather large. The number of chains W>2N is even larger. Due to this fact, the sampling process becomes inefficient and very costly (weeks using a parallel Matlab server). Worse, there are strong oscillations in the sampled fields due to the diagonal covariance choice that ignores spatial correlations. The resulting maximum a posteriori estimates and mean estimates of the density and velocity fields are not essentially flat with bumps in the reservoir areas as expected. These fields show some oscillations about the known background density and velocity fields, as seen in Figure 4. Still, the sample mean suggests the presence of regions of formed by different materials under the dome (see Figure 4). We have investigated this phenomenon further in one-dimensional setups. The oscillatory behavior observed in the MAP and mean fields increases. This seems to be related to the choice of a diagonal covariance and disappears under other choices. In the next section, we see how a different formulation which includes spatial correlations in the covariances improves the results.

### 5.2. Karhunen–Loève Expansions

In this section, we consider the corrections to the pressure and velocity fields as random fields. As previously detailed, in an infinite-dimensional setup [14], the goal of a Bayesian inverse problem is to estimate the posterior distribution

$$\begin{array}{c} \pi_{\mathrm{pt}}\left(d\xi \right)\propto L\left(\xi \right)\pi_{\mathrm{pr}}\left(d\xi \right) \end{array}$$

where ξ is a square-integrable function on a domain $\Omega \subset R^{n}$. Given a likelihood $L\left(\xi \right)\propto e^{\phi (\xi )}$ and a prior $\pi_{\mathrm{pr}}∼N\left(0,C\right)$, Karhunen–Loève expansions decompose random functions ξ drawn from Gaussian distributions N(0,C) as a linear combination of the eigenfunctions of *C* [14]

(19) $$\begin{array}{c} \xi =\sum_{\ell =1}^{\infty }\xi_{\ell }\phi_{\ell },\;\xi_{\ell }∼N\left(0,\lambda_{\ell }\right), \end{array}$$

associated to its eigenvalues $\lambda_{1}\geq \lambda_{2}\geq \ldots \geq \lambda_{\ell }\geq \ldots$. When ξ is a Gaussian random field with zero mean, the coefficients $\xi_{i}$ are independent Gaussians with mean zero and variances determined by the eigenvalues. The required eigenvalues and eigenvectors are easy to calculate for Matérn type covariance operators ${\left(\tau^{2}I-\Delta \right)}^{-\delta }$, where τ=1/l represents an inverse length scale and δ=γ+n/2 controls the sample regularity [31,52]. If $\mu_{i},\phi_{i}$ are the eigenvalues and eigenfunctions of $\tau^{2}-\Delta$ with homogeneous boundary conditions, the eigenvalues and eigenfunctions we seek are $\lambda_{i}=\mu_{i}^{-s}$, with −s=−δ/2=−(γ+n/2)/2 and $\phi_{i}$ (see [53,54]). The parameter γ controls the degree of irregularity. Gaussian and exponential kernels are particular cases of Matérn kernels. The exponential kernel corresponds to γ=1/2 and the Gaussian kernel to γ→∞.

In our framework, we can use zero Dirichlet or Neumann boundary conditions since we expect the correctors to vanish away from the dome. For a rectangle $\left[0,L_{x}\right]\times \left[0,L_{y}\right]$, the eigenvalues of $\left(\tau^{2}I-\Delta \right)$ with homogeneous Dirichlet boundary conditions are $\lambda_{q,r}=\tau^{2}+\pi^{2}\left(\frac{q^{2}}{L_{x}}+\frac{r^{2}}{L_{y}}\right)$ and the normalized eigenfunctions $\phi_{q,r}\left(x,y\right)=\frac{4}{L_{x}L_{y}}\sin\left(\frac{\pi qx}{L_{x}}\right)\sin\left(\frac{\pi ry}{L_{y}}\right)$, q,r≥1. Thanks to this fact, we can easily construct finite-dimensional approximations of the covariance operator as $C_{\Lambda }=P^{T}\Lambda P$ where Λ is a diagonal matrix formed by the selected largest eigenvalues and P a matrix whose columns are the corresponding discretized basis functions. Since we have some a priori information on the stratified medium, we can select some of the hyperparameters governing the Matérn covariances. The values of $\sigma_{\rho }^{2}$ and $\sigma_{v}^{2}$ are set equal to the known values of the variances of the density and velocity fields in the ambient medium. A value for the characteristic length l=1 is suggested by the known dimensions of the dome cap, under which we seek possible reservoirs. To exemplify the method, we will work with Matérn covariances for γ=1/2 and dimension n=2. We set γ=1/2 because it allows for irregular patterns, and it largely simplifies the calculation of the Matérn covariances due to the availability of explicit formulas for this kernel. More costly Bayesian formulations that include the hyperparameters as additional unknown variables to be sampled are proposed in [55], for instance.

In practice, we will use finite-dimensional approximations constructed as follows. Notice that ρ and $v_{p}$ in (15) must remain positive. We can enforce this constraint in two different ways. In the first, we choose a truncated multivariate normal distribution $p_{\mathrm{pr}}=N\left(0,C_{2N}\right)$ as a prior distribution when the resulting ρ and $v_{p}$ are positive and zero otherwise. Here, $C_{2N}$ is formed, combining two finite-dimensional approximations $C_{N}$ to the covariance operator *C*, one acting on $\nu_{\rho }$ and the other one on $\nu_{v}$. We relabel the basis functions as $\phi_{\ell }$ using the relation $\ell \left(q,r\right)=\left(q-1\right)N_{x}+r$, $1\leq q\leq N_{x}$, $1\leq r\leq N_{y}$. $N_{x}$ and $N_{y}$ represent the number of modes selected in each direction. Truncating the Karhunen–Loève expansions (19) of the random fields $\rho_{c}$ and $v_{p,c}$ to $N=N_{x}N_{y}$ modes and inserting

(20) $$\begin{array}{c} \rho_{\nu_{\rho }}=\sum_{\ell =1}^{N}\nu_{\rho ,\ell }\phi_{\ell },\;v_{p,\nu_{v}}=\sum_{\ell =1}^{N}\nu_{v,\ell }\phi_{\ell },\;\nu_{\rho ,\ell },\nu_{v,\ell }∼N\left(0,\lambda_{\ell }\right) \end{array}$$

in (15), we have an approximate parametric representation (18) of the density and velocity fields in the truncated model (4) in terms of the random variables $\nu =(\nu_{\rho },\nu_{v})$. We denote by F(ν) the observation operator (5) corresponding to the fields (18) with $\nu_{\rho }$ and $\nu_{v}$ given by (20).

The second strategy to enforce positivity constraints resorts to lognormal distributions, setting

(21) $$\begin{array}{c} \begin{array}{c} \rho \left(x,y\right)=\exp\left(\log\left(\rho \left(x,y\right)\right)\right),\;v_{p}\left(x,y\right)=\exp\left(\log\left(v_{p}\left(x,y\right)\right)\right), \end{array} \end{array}$$

where log(ρ(x,y)) and $\log(v_{p}\left(x,y\right))$ follow normal distributions. To determine the distribution parameters, notice that a distribution $\mathrm{lognormal}(\mu ,\sigma^{2})$ has mean $\mu_{0}=\exp\left(\mu +\sigma^{2}/2\right)$ and variance $\sigma_{0}=\exp\left(2\mu +\sigma^{2}\right)\left(\exp\left(\sigma^{2}\right)-1\right)$. Then, $\mu =\log\left(\rho_{0}\right)-\frac{1}{2}\log\left(\left(\sigma_{0}/\rho_{0}^{2}\right)+1\right)∼\log\left(\rho_{0}\right)-\frac{1}{2}\sigma_{0}/\rho_{0}^{2}$ (with $\mu ∼\log(\rho_{0})$ if $\sigma_{0}<<\rho_{0}^{2}$) and $\sigma^{2}=\log\left(\sigma_{0}/\rho_{0}^{2}\right)+1)$ (see [56]). In this case, we expand

(22) $$\begin{array}{c} \log\rho -\log\rho_{0}=\sum_{\ell =1}^{N}\nu_{\rho ,\ell }\phi_{\ell },\;\log v_{p}-\log v_{p,0}=\sum_{\ell =1}^{N}\nu_{v,\ell }\phi_{\ell }. \end{array}$$

We denote by F(ν) the observation operator (5) corresponding to the fields (21) and (22). The prior distribution is $N(0,C_{2N})$, formed by combining two finite-dimensional approximations $C_{N}$ to the covariance operator *C*, one acting on $\nu_{\rho }$ and the other one on $\nu_{v}$.

In either case, with these choices, the likelihood is given by (7) and the posterior distribution to be analyzed is given by (8). In view of the spatial correlations, we sample the resulting posterior probability (2) by means of Markov chain Monte Carlo methods, more specifically, a functional ensemble sampler (FES) (see [25]). This sampler follows a Metropolis-within-Gibbs approach that combines AIES for a subset of dominant modes that uses AIES on $E_{L}$ the low-wavenumber KL components and the preconditioned Crank–Nicolson sampler (pCN) on the high-wavenumber KL components. This method remains stable as we increase dimensionality, that is, the number of total modes we keep. If $E_{L}=0$, the method is just pCN. However, for small positive $E_{L}$ it may converge faster, though it is quite difficult to tune adequate values of $E_{L}$. We have adapted this algorithm to our setup (see Appendix A for details). In our tests, we have initialized the chains sampling values $\nu^{i}$, i=1,…W, W>4N+1, with probability $p_{\mathrm{pr}}$.

**Figure 5:**
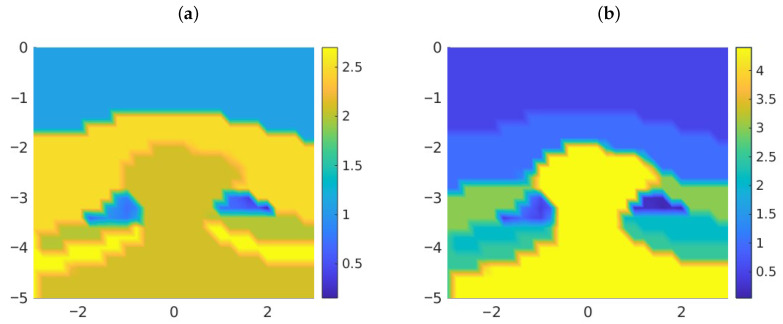
(**a**) Density ρ and (**b**) velocity fields $v_{p}$ for the salt dome geometry in Figure 1c after 60 steps of the FES algorithm with 400 modes.

**Figure 6:**
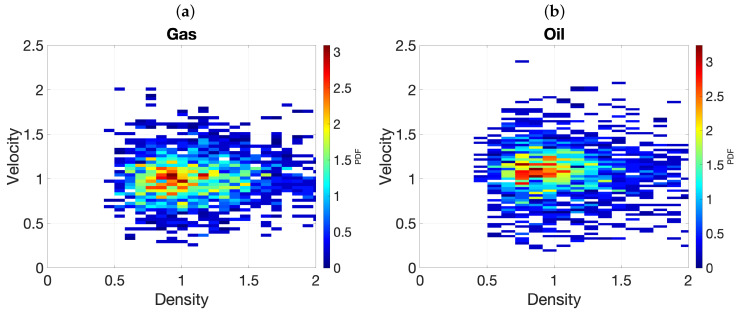
Density–velocity histogram projections constructed from samples in the oil and gas regions.

Figure 5 illustrates the results. Notice that inclusions of different materials are identified under the salt dome cap, with spatial variations from top to bottom, in these visualizations of the MAP estimates. These deposits are difficult to identify by standard seismic imaging techniques due to the screening effect of the dome cap. From the theoretical point of view, this approach is more stable that the Fourier approach. Well-posedness and stability as the number of modes tends to infinity can be proved in the framework developed in [14] (see also [55]). However, uncertainty about the details of the deposits is still large. For the MAP estimates, the values of the densities and the velocities in the dome region range in the intervals [0.4369,0.8655] and [0.8326,1.5136] (respectively) for oil and [0.4204,0.8035] and [0.3329,0.6595] (respectively) for gas. For the mean estimates, the values of the densities and the velocities in the dome region range in the intervals [0.3861,0.9026] and [0.3679,2.4381] (respectively) for oil and [0.5406,1.0403] and [0.3679,2.7183] (respectively) for gas. Figure 6 represents density-velocity histograms constructed from the sample values in the oil and gas regions. Moreover, the results are strongly dependent on the prior covariances. In practice, the covariance parameters are unknown, and they should be adjusted through a more complex Bayesian formulation that includes them as additional unknown hyperparameters with their own probability distribution [55]. Nevertheless, the question arises of whether Mátern covariances are a good choice for this particular problem [34,35].

## 6. Discussion

We have analyzed different strategies to quantify the uncertainty in inverse scattering problems by means of inverse Bayesian formulations. To this end, we assume we have previous information on the background medium. The goal is to determine the presence of possible anomalies or inclusions in the medium and to characterize them with quantified uncertainty. We focus on imaging applications that employ time-dependent waves, though our techniques are applicable to other imaging setups. In the posterior probability, the likelihood compares the recorded data to the observed values for the proposed anomalies/inclusions as defined by a forward model of wave propagation. Our tests use synthetic data, as detailed at the end of Appendix A.

In homogeneous media, topological energy methods [13] are known to provide information on the number of anomalies and their approximate location and size, but not on their nature. Working with star-shaped parameterizations, anomalies can be characterized by a low number of random variables. In shear elastography for tissues, Bayesian formulations with topological energy priors for the number and shape of the anomalies, but no prior information on their nature, allow us to extract their shape and material constants with quantified uncertainty from the posterior probability. Tissues are somewhat special because we can approximate their density by a constant everywhere. Anomalies are then characterized by their shear modulus. In stratified media, imaging methods such as migration techniques [42] provide a priori information on the overall layered soil structure. We are interested here in obtaining precise details on the size, orientation, location, and nature of inclusions. Again, assuming no a priori information on the nature of the inclusion, the posterior probability yields information on its nature. However, inclusions now are characterized by two material parameters, density and velocity. This results in an increased uncertainty in the predictions, with several high probability configurations corresponding to different shapes and velocities. Different MCMC tools are adequate depending on the circumstances [21]. In both cases, we have sampled the posterior probability by an affine invariant ensemble MCMC sampler (AIES). These samplers can be parallelized and are efficient for posterior distributions involving few and poorly scaled unknowns [24], even when they are multimodal. In the tests carried out here, the main mode captures the true anomalies/inclusions. These tests use synthetic data generated for incident waves of a single frequency. Combining several frequencies we might be able to reduce uncertainty, suppressing multiple high probability modes. The rationale behind this conjecture is that spurious modes often vary with the frequency. By including data from different frequencies, we may strengthen the main mode (represented in all frequency data) and dilute the variable secondary modes. This phenomenon has already been observed in topological studies [57].

The previous approach is low-dimensional and low in cost. We use prior information on the geometry of inclusions but not on their nature. Often, we ignore that information, and we have to increase dimensionality to capture the details we need. To this end, we represent densities and velocities by random fields and characterize the deposits by analyzing the spatial variation in these fields. We chose to include the available information on the density and velocity of the underlying stratified medium in the direct problem for wave propagation and parameterize the correction, with zero prior information about it. Unlike previous work that parametrizes the fields in terms of their values on a mesh [12], we resort here to global expansions in terms of two different bases. Both approaches lead to Bayesian formulations for a finite number of random variables. In the first one, we represent the corrector fields by truncated Fourier series expansions. The coefficients define a finite collection of random variables for which we formulate the Bayesian inverse problem. In the second one, we represent the corrector fields by random fields, introduce their Karhunen–Loève (KL) expansions and approximate the fields by truncated KL expansions, in terms of a finite number of random variables. This approach can rely on lognormal distributions to ensure the positivity of the resulting densities and velocities. In both cases, we include zero a priori information of the correctors. A delicate point is the choice of the prior covariance since the results depend strongly on this choice. From the theoretical point of view, the KL approach has a number of advantages: it remains stable as the number of modes kept in the truncated expansion grows and the posterior probabilities can be sampled by preconditioned Crank-Nicholson methods (pCN) or functional ensemble (FES) methods in a robust way [25].

We have tested both approaches on a salt dome configuration. We assume we have characterized the main stratified structure by other techniques but lack details on the composition under the dome cap, due to its screening effect. In particular, we ignore whether oil/gas reservoirs are present. The Fourier strategy barely suggests the presence of deposits under the dome cap. Moreover, the choice of a diagonal covariance allows for unexpected oscillatory behaviors. Instead, the KL strategy relies on matrix covariances that enforce some spatial correlation or definite behaviors on the corrector fields. We have sampled the posterior probability by means of functional ensemble methods, which employ AIES on a small number of modes and pCN on the rest. In this way, we have been able to visualize the presence of reservoirs at the sides of the salt dome. Although the values of the fields obtained are far from the actual values, it is important to mention that despite the initial information on the unknown parameters being zero and the presence of the salt dome attenuating the received signal, it is still possible to identify materials distinct from those of the background fields. Notice that we use zero prior information on the deposits, the results might improve if we add some information or resort to the procedure explained in Section 4. The choice of the covariance matrix is a delicate issue too. To exemplify the procedure, we have used Matérn covariances with ad-hoc hyperparameter choices. That is a restricted class, different covariances are used for other applications [34,35]. We lack a basis to choose a specific type of covariance. On the other hand, the results might improve by considering the covariance hyperparameters as additional unknowns to be sampled [55], which would lead to a more complex Bayesian formulation and a more costly sampling strategy.

Our work shows that Bayesian inversion techniques have a great potential for improving imaging techniques, by allowing us to obtain the most probable solutions, and by quantifying uncertainty in terms of other probable configurations as well as the uncertainty range in the predictions. Starting with simple low-dimensional configurations, we have progressed to study configurations of practical interest in high dimensions. Our study also highlights the importance of having sharp a priori information (which can come from other imaging techniques or previous data studies) and choosing the covariances appropriately.

## Figures and Tables

**Table 1:** Dimensional parameters used in the tissue simulations.

*L*	*T*	$\rho_{i}$	ρ	$\mu_{i}$	μ	$c_{i}$	*c*	$f_{M}$	$f_{0}$
0.01 m	0.01 s	ρ	$10^{3}\;\frac{\mathrm{kg}}{m^{3}}$	16 kPa	1.69 kPa	$4\;\frac{m}{s}$	$1.3\;\frac{m}{s}$	50 Hz	$\frac{\rho L}{T^{3}}$

**Table 2:** Dimensional parameters used in simulations in stratified media.

*L*	*T*	$\rho_{i}$	$\rho_{0}$	$v_{p,i}$	$f_{M}$	$f_{0}$
1 km	1 s	$2.1\rho_{0}$	$1000\;\frac{\mathrm{kg}}{m^{3}}$	$4.4\;\frac{\mathrm{km}}{s}$	2 Hz	$100\frac{\rho_{0}L}{T^{3}}$

## Data Availability

The paper contains the parameter values necessary to generate synthetic data by solving the forward model and adding noise as explained. In this way, the results are reproducible. The specific realizations employed here could be available upon reasonable request.

## Appendix A. AIES and FES Samplers

We next summarize the adapted FES algorithm to sample a posterior probability $p_{\mathrm{pt}}\left(\nu \right)$, $\nu =(\nu_{\rho },\nu_{v})$ when $\nu_{\rho }$ and $\nu_{v}$ follow distributions $N(0,C_{\rho })$ and $N(0,C_{v})$. Set C equal to a block diagonal matrix with diagonal blocks $C_{\rho }$ and $C_{v}$. We calculate the eigenvalues and eigenfunctions for the KL expansions and order them in a decreasing order. Then, we store the first $E_{L}$ eigenvalues and form matrices $J_{\rho }$ and $J_{v}$ with their ordered column eigenvectors.

**Algorithm A1** FES Sampler
1: **Step 1:** Set the size *N* of $\nu_{\rho }$ and $\nu_{v}$ and the number of eigenvalues kept for each $E_{L}<N$. Compute $P_{\rho }=J_{\rho }J_{\rho }^{T}$ and $P_{v}=J_{v}J_{v}^{T}$. Set P equal to a block diagonal matrix with diagonal blocks $P_{\rho }$ and $P_{v}$. Set Q=I−P. 2: **Step 2:** Select the number of chains *W* and steps $I_{\max}$. Initialize $\nu_{w}$ sampling $N(0,C_{\rho })$ and $N(0,C_{v})$ for w=1,…,W, $W>4E_{L}$, to generate the two blocks of components. Set the sampling parameters a=2 and ω=0.02. 3: **Step 3:** Calculate the associate densities and velocities $\rho (\nu_{w})$ and $v_{p}\left(\nu_{w}\right)$, by adding the parameterized corrections to their background values according to the selected parameterization, for w=1,…,W. 4: **Step 4:** Calculate the logarithm of the prior distribution for each *w*: ${\mathrm{logprior}}_{w}=-\frac{\nu_{w}^{T}C^{-1}\nu_{w}}{2}.$ 5: **Step 5:** Solve the forward wave problem with fields $\rho (\nu_{w})$ and $v_{p}\left(\nu_{w}\right)$ for w=1,…,W and evaluate the observation operator $F(\nu_{w})$. 6: **Step 6:** Calculate the logarithm of the likelihood for w=1,…,W: ${\mathrm{loglike}}_{w}=-\frac{∥F\left(\nu_{w}\right){-d∥}_{2}^{2}}{2\sigma_{n}^{2}}.$ 7: **Step 7:** Calculate the logarithm of the posterior probability fpr w=1,…,W $$\begin{array}{c} {\mathrm{logpost}}_{w}={\mathrm{logprior}}_{w}+{\mathrm{loglike}}_{w}. \end{array}$$ 8: **Step 8:** Initialize $\nu_{{\mathrm{prev}}_{w}}$, ${\mathrm{logpostprev}}_{w}$ and ${\mathrm{logpriorprev}}_{w}$ as $\nu_{w}$, ${\mathrm{logpost}}_{w}$ and ${\mathrm{logprior}}_{w}$. 9: **Step 9:** Apply FES 10: **for** i=1 **to** $I_{\max}$ **do** 11: **Step 9.1:** Apply AIES to the selected first $E_{L}$ modes in each block 12: **Step 9.1.1:** Generate a permutation per of (1,2,3,…,W) without fixed sites. 13: **Step 9.1.2:** Generate random numbers and vectors: 14: vecz $={\left((a-1)\times \mathrm{randw}+1\right)}^{2}/a$, and logvecz =log(vecz), where randw is a vector formed by W random numbers U(0,1) and operations are performed component wise. 15: **Step 9.1.3:** Perform the cycle 16: **for** w=1 **to** *W* **do** 17: **Step 9.1.3.1:** Propose the update $$\nu_{\mathrm{prop},w}=\nu_{\mathrm{prev},w}+\left(1-{\mathrm{vecz}}_{w}\right)\;P\;\left(\nu_{\mathrm{prev},\mathrm{per}(w)}-\nu_{\mathrm{prev},w}\right)$$ 18: **Step 9.1.3.2:** Calculate ${\mathrm{logprior}}_{w}=-\frac{\nu_{w}^{T}C^{-1}\nu_{w}}{2}.$ 19: **Step 9.1.3.3:** Generate ${\mathrm{logr}}_{w}$ as log(U(0,1)). 20: **if** ${\mathrm{logr}}_{w}<{\mathrm{logpriorprop}}_{w}-{\mathrm{logpostprev}}_{w}+{\mathrm{logvecz}}_{w}\times \left(2E_{L}-1\right)$ **then** 21: **Step 9.1.3.3.1:** Solve the forward wave problem with fields $\rho (\nu_{w})$ and $v_{p}\left(\nu_{w}\right)$ for w=1,…,W and evaluate the observation operator $F(\nu_{w})$. 22: **Step 9.1.3.3.2:** Calculate ${\mathrm{loglikeprop}}_{w}=-\frac{∥F\left(\nu_{\mathrm{prop},w}\right){-d∥}_{2}^{2}}{2\sigma_{n}^{2}}.$ 23: **Step 9.1.3.3.3:** Calculate ${\mathrm{logpostprop}}_{w}={\mathrm{logpriorprop}}_{w}+{\mathrm{loglikeprop}}_{w}.$ 24: **Step 9.1.3.3.4:** Calculate the logarithm of the acceptance probability α $$\log\alpha ={\mathrm{logpostprop}}_{w}-{\mathrm{logpostprev}}_{w}+{\mathrm{logvecz}}_{w}\times \left(2E_{L}-1\right)$$ 25: **Step 9.1.3.3.5:** Accept or reject the proposal. Generate ${\mathrm{logr}}_{w}$ as log(U(0,1)). 26: **if** ${\mathrm{logr}}_{w}<\log\left(\alpha \right)$ **then** 27: $\nu_{\mathrm{prev},w}=\nu_{\mathrm{prop},w}$ 28: ${\mathrm{logpostprev}}_{w}={\mathrm{logpostprop}}_{w}$ 29: ${\mathrm{loglikeprev}}_{w}={\mathrm{loglikeprop}}_{w}$ 30: **end if** 31: **end if** 32: **end for** 33: **Step 9.2:** Apply pCN to the remaining modes in each block 34: **for** w=1 **to** *W* **do** 35: **Step 9.2.1:** Propose the update $$\nu_{\mathrm{prop},w}=P\;\nu_{\mathrm{prev},w}+Q\;\left(\sqrt{1-\omega^{2}}\;\nu_{\mathrm{prev},w}+\omega \;\xi_{w}\right),$$ where $\xi_{w}∼N\left(0,C\right)$. 36: **Step 9.2.2:** Solve the forward wave problem with fields $\rho (\nu_{w})$ and $v_{p}\left(\nu_{w}\right)$ for w=1,…,W and evaluate the observation operator $F(\nu_{w})$. 37: **Step 9.2.3:** Calculate ${\mathrm{loglikeprop}}_{w}=-\frac{∥f\left(\nu_{\mathrm{prop},w}\right)-d^{\mathrm{obs}}{∥}_{2}^{2}}{2\sigma_{\mathrm{noise}}^{2}}.$ 38: **Step 9.2.4:** Calculate the logarithm of the acceptance probability $$\log\alpha ={\mathrm{loglikeprop}}_{w}-{\mathrm{loglikeprev}}_{w}.$$ 39: **Step 9.2.5:** Accept or reject the proposal. Generate ${\mathrm{logr}}_{w}$ as log(U(0,1)). 40: **if** ${\mathrm{logr}}_{w}<\log\left(\alpha \right)$ **then** 41: Calculate ${\mathrm{logpriorprop}}_{w}=-\frac{\nu_{\mathrm{prop},w}^{T}C^{-1}\nu_{\mathrm{prop},w}}{2},$ 42: $\nu_{\mathrm{prev},w}=\nu_{\mathrm{prop},w}$, 43: ${\mathrm{loglikeprev}}_{w}={\mathrm{loglikeprop}}_{w}$, 44: ${\mathrm{logpostprev}}_{w}={\mathrm{loglikeprop}}_{w}+{\mathrm{logpriorprop}}_{w}$. 45: **end if** 46: **end for** 47: **end for**

As we run the algorithm, we store the moves for the *W* chains along $I_{\max}$ steps. Discarding *B* initial samples for each chain as a burn-up stage, the remaining collection of vectors sample the posterior probability. As said earlier, value $p_{0}$ is not needed because the employed ratio cancels it. Gelman–Rubin tests [58] are used to test convergence.

To sample with AIES, we set the number of modes equal to the number of parameters for $\nu_{\rho }$ and $\nu_{v}$, that is, $E_{L}=N$, and skip the pCN part. To accelerate the process, we choose educated starting values for the chains $\nu_{w}$ that correspond to functions with zero or one or two bumps at each side of the lower part of the dome cap. The number of bumps is randomly selected at each side. The location of their center is randomly selected in a box enclosing the lower part of the dome, as well as their radius. Their heights are randomly generated in $\left[0,\frac{\max(\rho )-\min(\rho )}{2}\right]$ and $\left[0,\frac{\max\left(v_{p}\right)-\min\left(v_{p}\right)}{2}\right]$, considering the known background values. We keep the choices for which the posterior probability is smaller than the probability of the configuration without oil and gas traps. To sample with pCN we set $E_{L}=0$ and skip the AIES part. To sample with FES, we choose $E_{L}<N$.

Notice that the use of logarithms becomes necessary because the exponentials of negative values can be so small as to be interpreted as zero by a computer, rendering the procedure useless. Therefore, the original algorithm is implemented taking the logarithms of the amounts to be compared or processed.

In the tests presented here, we have employed synthetic data d generated by numerically solving the forward wave model and corrupting with noise. For completeness, we detail the procedure in Algorithm A2.

**Algorithm A2** Generation of synthetic data
1: **Step 1:** Create a spatial mesh in the computational region [ax,bx]×[ay,by] adapted to the true subsurface configuration. 2: **Step 2:** Create a temporal mesh in the time interval [0,T]. 3: **Step 3:** Define the locations of sources and receivers, and the recording times (independent of the previous two meshes). 4: **Step 4:** Generate the background reference density and velocity fields, and construct the corresponding FEM matrices for the selected spatial mesh. 5: **Step 5:** Approximate the solution of the forward wave problem by finite difference discretization in time and finite element discretization in space. 6: **Step 6:** Generate the true data at the receivers by evaluating the numerical solution at the required times and locations. 7: **Step 7:** Generate the synthetic noisy data d by adding noise of magnitude α.

We can use the formula mentioned in Section 4.2 to characterize the noise, though other choices are possible.
